# Visual acuity outcome of stable proliferative diabetic retinopathy following initial complete panretinal photocoagulation

**DOI:** 10.1136/bmjophth-2022-001068

**Published:** 2022-09-29

**Authors:** Dun Jack Fu, Sridevi Thottarath, Livia Faes, Konstantinos Balaskas, Pearse A Keane, Dawn Sim, Sobha Sivaprasad

**Affiliations:** NIHR Biomedical Research Centre, Moorfields Eye Hospital NHS Foundation Trust, London, UK

**Keywords:** retina, treatment lasers

## Abstract

**Background:**

Recent clinical trials on proliferative diabetic retinopathy (PDR) show that presenting visual acuity can be stabilised with panretinal photocoagulation (PRP) within 2 years despite the need for supplementary PRP or anti-vascular endothelial growth factor therapy for concomitant diabetic macular oedema (DMO). It is unclear whether similar results can be obtained in daily clinical practice. Here, we query the probability of vision loss in patients with treatment-naïve PDR who have attained stability after PRP and its predictors.

**Methods:**

Retrospective cohort study at a tertiary eye centre between 01 January 2015 and 31 December 2019, wherein 2336 eyes met study criteria with first record of stable PRP-treated PDR in at least one eye. Kaplan-Meier and Cox proportional hazards modelling were used to report the probability of vision loss of at least five Early Treatment Diabetic Retinopathy Study (ETDRS) letters.

**Results:**

The probability of losing at least five ETDRS letters was 50% at 3.32 (95% CI, 2.94 to 3.78) years after achieving first stability post PRP in treatment-naïve PDR. The mean decrease at this event was 14.2 (SD 13.0) ETDRS letters irrespective of the presence of DMO. The strongest risk factor for vision loss was a history of DMO at baseline (HR 1.62 (95% CI, 1.34 to 1.95), p<0.001).

**Discussion:**

One in two patients with stable treated PDR lose a line of vision by 3.5 years. This resulted in 15% of patients losing their eligibility to drive. Notably, 13% of the cohort died during the follow-up period.

**WHAT IS ALREADY KNOWN ON THIS TOPIC**

- Proliferative diabetic retinopathy (PDR) is treated with panretinal photocoagulation, but it is unclear whether visual loss can occur after disease stabilisation in real-world data.

**WHAT THIS STUDY ADDS**

- Despite achieving stability for PDR, the majority of patients proceed to lose clinically meaningful vision.

**HOW THIS STUDY MIGHT AFFECT RESEARCH, PRACTICE OR POLICY**

- Patients with PDR require further management even after achieving ‘stability’ and macular ischaemia as a contributory cause for deterioration of vision loss ought to be further researched.

## Introduction

Proliferative diabetic retinopathy (PDR) is characterised by the growth of abnormal new vessels on the retina and/or optic disc. These clinical features alone are not usually associated with visual impairment unless there is coexistent diabetic macular oedema (DMO).^1^ Anti-vascular endothelial growth factor (anti-VEGF) therapy is the treatment of choice for DMO. The Diabetic Retinopathy Clinical Research (DRCR) Protocol T trial reported that less than 5% of patients with DMO treated with anti-VEGF therapy lost 10 or more Early Treatment Diabetic Retinopathy Study (ETDRS) letters by the end of 2 years.^2^ In clinical practice, treatment naïve eyes with low or high-risk PDR that does not require vitreoretinal surgery but have coexistent DMO are treated with a combination of anti-VEGF therapy and panretinal photocoagulation (PRP).^3 4^ There is limited literature on the probability of visual loss in patients presenting with PDR with and without DMO over time in real life.^5–7^

The main cause of severe visual loss in PDR is due to complications of untreated or suboptimally treated PDR such as vitreous haemorrhage and tractional retinal detachment. The landmark clinical trials, Diabetic Retinopathy Study^8^ and the ETDRS^5^ demonstrated that PRP in patients with PDR reduces the risk of severe visual loss by 50%. Eyes with non-clearing vitreous haemorrhage or tractional retinal detachment threatening or involving the macula may require vitrectomy.^9^ Another rarer complication of PDR that can cause severe visual loss is neovascular glaucoma.^10^ In the UK, where diabetic retinopathy screening is well-established, most patients who present with PDR have low-risk characteristics and a small proportion present as high-risk PDR and do not usually require prompt vitrectomy. Very few patients with PDR present with neovascular glaucoma.^11^ Over the last 40 years, PRP has prevented severe visual loss in most patients who present early with PDR. However, there are recent reports that show the proportion lost to follow-up is high in patients with PDR and cost of treatment may be a factor.^5–7^ Contemporary data on long-term visual outcome of treatment naïve patients who present with low or high-risk PDR treated with initial complete PRP in a fully public-funded health system is lacking.

Recent studies such as Protocol S and the CLARITY trial, designed to examine the role of anti-VEGF therapy as an alternative therapy to PRP for active PDR not requiring vitreoretinal surgery at baseline, showed that eyes treated with PRP maintain presenting visual acuity (VA) over 1–2 years.^12 13^ Approximately 45% required further fill-in PRP over 2 years.^1^ Moreover, in the PRP arm of Protocol S, 34% developed vitreous haemorrhage, 10% had retinal detachment and 15% underwent vitreoretinal surgery by 2 years.^1^ The 5-year follow-up of Protocol S showed stable visual outcomes with PRP in approximately 60% of the initial study cohort who were retained in the study.^14^ Factors that determine visual outcomes in Protocol S have been explored but they explain only one-third of the variations.^15^ The predictors of visual outcome of patients who achieve first stabilisation of PDR in clinical practice is unclear. These findings are consistent with retrospective studies. Indeed, a large Indian multicentre study described that about 55% of patients with stable treated PDR had a VA measure of ≥6/12 Snellen in a 10 years follow-up.^16^ Moreover, Kaiser and colleagues similarly found in their cohort study that of the 76% of patients with PDR presenting with 6/12 or more Snellen VA could maintain this level of vision 1 year after PRP.^17^

The aim of this study was to assess the probability of visual loss over time of patients from first documentation of stable PDR after PRP in routine clinical practice and to determine the predictors of visual loss. We considered outcomes in eyes with and without DMO at presentation.

## Methods

### Study design and setting

This retrospective cohort study was conducted at Moorfields Eye Hospital National Health Service Foundation Trust, a tertiary centre in London, UK. The study was conducted in compliance with the Declaration of Helsinki and reported in accordance with the Strengthening the Reporting of Observational Studies in Epidemiology reporting guideline. Informed consent from the study cohort was not required as per the standard when using retrospective, de-identified data for research within the UK National Health Service.

### Cohort

The cohort comprised consecutive patients with a first record of stable PDR (coded R3S) after treatment with PRP with or without history of previous vitrectomy in at least one eye between 1 January 2015, and 31 December 2019. Patients were required to have at least two records of VA beyond 3 months of first R3S record. Stability was defined by the retinal specialist after clinical examination, which may or may not include retinal photographs. In clinical practice, stability is achieved if the neovascularisation has completely regressed or partially regressed compared with previous visit or remain unchanged despite adequate laser coverage of the retina. The cohort included both phakic and pseudophakic eyes, as well as eyes with and without DMO. Baseline DMO status was subcategorised as eyes with absence of DMO (coded M0), active DMO on anti-VEGF treatment (coded M1A) and eye with stable DMO under observation (M1S). The M1S eyes may have parafoveal oedema or centre-involving oedema with good VA or post-treated DMO that is persistent but stable. If both eyes had a record of stable treated PDR, inter-eye correlation was acknowledged and considered by selecting one eye at random using the sample function of base R software, V.3.6.2 (R Foundation for Statistical Computing). Exclusion criteria were study eye with: active PDR or any other retinopathy grades in the study eye that have not achieved stability; incomplete baseline records; fewer than two post-baseline records of VA; less than 3 months of follow-up data; ungradable maculopathy status.

#### UK diabetic eye screening programme gradings

The nationwide diabetic eye screening programme invites all patients with diabetes aged 12 years or over to annual primary care-based screening. Here, two-field fundus photography (one image centred on the macula and a second image centred on the optic disc) is acquired and graded according to the English Screening Programme for Diabetic Retinopathy standards.^18^ Briefly, retinopathies are graded into four levels: none (R0), background (R1; microaneurysms, retinal haemorrhages, venous loops or any exudate in the presence of other non-referable features), pre-proliferative (R2; venous beading, reduplication, multiple blot haemorrhages or intraretinal microvascular abnormality) and proliferative (R3) retinopathy. R3 is further classified into active proliferative disease (R3A; new vessels at the disc, elsewhere, pre-retinal or vitreous haemorrhages or pre-retinal fibrosis with or without tractional detachment) and stable treated proliferative disease (R3S). Maculopathy and photocoagulation are graded as absent (M0, P0) or present (M1, P1). M1 includes the presence of exudates within one disc diameter of the centre of the fovea, retinal thickening within one disc diameter of the centre of the fovea, a group of exudates within the macula or any microaneurysm or haemorrhage within one disc diameter of the centre of the fovea only associated with a best VA of 20/40 Snellen or below. When gradings cannot be assigned due to image quality, ungradable (U) is assigned.

### Study outcomes

The primary outcome was time to a decrease of at least five ETDRS letters (one Snellen line) from baseline recorded at two consecutive visits post stability for the whole cohort. These outcomes were also stratified based on DMO status (M0, M1A or M1S). Secondary outcomes included: (a) predictors of a loss of five letters and (b) incident ineligibility to meet the UK legal limit of driving after achieving stability of PDR (ie, VA <70 ETDRS letters or 6/12 Snellen in both eyes) and (c) outcome of vitrectomised eyes and (d) patients with missing data defined as loss to follow-up. As recent treatment of DMO or vitrectomy might influence final visual outcome, we repeated the primary outcome analysis only in eyes who did not have records of anti-VEGF therapy or supplementary PRP or vitrectomy at least 6 months prior to two consecutive records of five-letter loss. The data of a random sample of 100 patients with loss of at least five letters and the optical coherence tomography (OCT) scans were examined to ensure reliability of the electronic medical records.

### Statistical analysis

All data analyses were carried out with R (V.3.5.1).^19^ Hazards were modelled with Kaplan-Meier models.^20^ Survival curves were plotted using the classical Kaplan-Meier estimator based on tabulation of the number at risk and number of events at each unique event time. For stratified curves, averages for subpopulations were fitted for each of these models to plot the cumulative hazard function with each grouping variable.

For covariable effects, multivariable Cox proportional hazards models^21^ were used to relate visual outcomes to clinical co-variables including VA at baseline; history of previous DMO or concomitant presence of DMO at baseline; previous vitrectomy; pseudophakic eyes. Confounding variables were included as independent co-variables. Subgroup analysis was done to assess outcomes of vitrectomised eyes. Two-sided p values were reported, and p<0.05 was considered significant. Mean (SD) values were reported unless otherwise specified. All clinical data were recorded within an electronic medical record application (OpenEyes Foundation), as previously described.^22^

### Patient and public involvement

Due to the post-hoc analysis methodology of this study, it was not appropriate or possible to involve patients or the public in the conduct of our research.

## Results

### Cohort demographics and clinical features

Between 01 January 2015 and 31 December 2019, a total of 2336 patients with stable R3S recorded for at least one eye met the inclusion criteria (see online supplemental eFigure 1). A total of 1312 (56.2%) had no DMO at baseline (M0) and 373 (16.0%) and 651 (27.8%) had M1A and M1S, respectively. The mean age of the cohort was 59.3 (SD 13.9) years and 1406 (60.2%) were men. The cohort was multiethnic and 35.9% of patients with a record of ethnicity data were whites (table 1). The mean VA of the whole cohort was 66.2 (SD 21.4) ETDRS letters (Snellen equivalent, 20/50) (table 2), wherein 65.2% were ≥70 letters. Of those with baseline VA of ≥70 letters (Snellen equivalent, ≥20/40), 592 (38.9%) had either active or stable DMO. A total of 244 (10.4%) had baseline VA of ≤35 letters (Snellen equivalent, 20/200), of which 51.6% had DMO. The cohort included 199 (8.5%) patients with history of vitrectomy and 275 (11.8%) were pseudophakic.

10.1136/bmjophth-2022-001068.supp1 Supplementary data

**Table 1:** Demographics of cohort

	M0 (N=1312)	M1A (N=373)	M1S (N=651)	Overall (N=2336)
Year of first achieving stability of PDR (R3S)				
2015	364 (27.7%)	78 (20.9%)	148 (22.7%)	590 (25.3%)
2016	253 (19.3%)	83 (22.3%)	138 (21.2%)	474 (20.3%)
2017	222 (16.9%)	78 (20.9%)	125 (19.2%)	425 (18.2%)
2018	249 (19.0%)	76 (20.4%)	126 (19.4%)	451 (19.3%)
2019	224 (17.1%)	58 (15.5%)	114 (17.5%)	396 (17.0%)
Gender				
Female	533 (40.6%)	140 (37.5%)	257 (39.5%)	930 (39.8%)
Male	779 (59.4%)	233 (62.5%)	394 (60.5%)	1406 (60.2%)
Age				
Mean (SD)	59.0 (14.5)	59.7 (13.1)	59.8 (13.2)	59.3 (13.9)
Median (min, max)	59.8 (21.0, 90.8)	60.1 (25.6, 91.8)	60.3 (22.7, 87.6)	60.1 (21.0, 91.8)
Ethnicity				
Caucasian	333 (25.4%)	99 (26.5%)	138 (21.2%)	570 (24.4%)
Afro Caribbean	169 (12.9%)	44 (11.8%)	75 (11.5%)	288 (12.3%)
South Asian	385 (29.3%)	109 (29.2%)	202 (31.0%)	696 (29.8%)
Chinese	5 (0.4%)	0 (0%)	5 (0.8%)	10 (0.4%)
Mixed	10 (0.8%)	4 (1.1%)	7 (1.1%)	21 (0.9%)
Unknown	410 (31.2%)	117 (31.4%)	224 (34.4%)	751 (32.1%)

Baseline demography characteristic shown for cohort at baseline that is, first recording of stable proliferative diabetes following panretinal photocoagulation. Data shown for entire cohort (overall), as well as stratified by macular status: absence of diabetic macular oedema (DME; M0), active DME on treatment (M1A) or stable DME under observation (M1S). Mean, SD, median, minimum and maximum values are shown for age in years. Proportions in percentages shown for gender, ethnicity and year of first record of stability.

PDR, proliferative diabetic retinopathy.

**Table 2:** Baseline clinical features of study eyes

	M0 (N=1312)	M1A (N=373)	M1S (N=651)	Overall (N=2336)
Baseline VA				
Mean (SD)	68.3 (21.2)	61.9 (19.7)	64.6 (22.3)	66.2 (21.4)
Median (min, max)	76.0 (0, 94.0)	70.0 (0, 89.0)	70.0 (0, 94.0)	76.0 (0, 94.0)
Baseline VA by category				
≥70	931 (71.0%)	188 (50.4%)	404 (62.1%)	1523 (65.2%)
51–69	190 (14.5%)	107 (28.7%)	114 (17.5%)	411 (17.6%)
36–50	73 (5.6%)	35 (9.4%)	50 (7.7%)	158 (6.8%)
≤35	118 (9.0%)	43 (11.5%)	83 (12.7%)	244 (10.4%)
Lens status				
Phakic	1176 (89.6%)	318 (85.3%)	567 (87.1%)	2061 (88.2%)
Pseudophakic	136 (10.4%)	55 (14.7%)	84 (12.9%)	275 (11.8%)
Previous vitrectomy				
No	1201 (91.5%)	332 (89.0%)	604 (92.8%)	2137 (91.5%)
Yes	111 (8.5%)	41 (11.0%)	47 (7.2%)	199 (8.5%)
Previous DME				
No	1266 (96.5%)	263 (70.5%)	582 (89.4%)	2111 (90.4%)
Yes	46 (3.5%)	110 (29.5%)	69 (10.6%)	225 (9.6%)

Baseline (first recording of stable proliferative diabetes following panretinal photocoagulation) visual acuity (VA) in Early Treatment Diabetic Retinopathy Study letters shown for overall cohort and substratified by macular status. Mean, median, SD, minimum and maximum values shown. Lens status, as well as, history of vitrectomy or diabetic macular oedema is also shown.

DME, diabetic macular oedema.

### Proportion of patients losing one Snellen line of vision (ETDRS ≥5 letters)

A total of 1236 (52.9%) eyes recorded loss of five ETDRS letters or more from baseline confirmed at two consecutive visits during the observation period, with the median decrease in VA from baseline being nine letters (table 3A). Of note, this event signified a drop in total VA below 70 letters in the study eye of 719 (58.2%) patients, of which 377 were equal to or above 70 letters at baseline (table 3B).

**Table 3:** Eyes that lost at least five ETDRS letters

(a)	M0 (n=678)	M1A (n=206)	M1S (n=356)	Overall (n=1240)
Baseline VA (mean; ETDRS letters)				
Mean (SD)	72.7 (13.3)	67.5 (14.2)	69.3 (16.8)	70.9 (14.7)
Median (min, max)	76.0 (11.0, 94.0)	70.0 (11.0, 89.0)	76.0 (10.0, 94.0)	76.0 (10.0, 94.0)
Change in VA from baseline (mean; ETDRS letters)				
Mean (SD)	−14.9 (14.2)	−13.7 (11.5)	−13.1 (11.2)	−14.2 (13.0)
Median (min, max)	−9.00 (−83.0, –5.00)	−9.00 (−63.0, –5.00)	−9.00 (−85.0, –5.00)	−9.00 (−85.0, –5.00)
Proportion >70 ETDRS letters in study eye				
No	372 (54.9%)	142 (68.9%)	209 (58.7%)	723 (58.3%)
Yes	306 (45.1%)	64 (31.1%)	147 (41.3%)	517 (41.7%)
Proportion <70 ETDRS letters in both eyes				
No	504 (74.3%)	128 (62.1%)	240 (67.4%)	872 (70.3%)
Yes	174 (25.7%)	78 (37.9%)	116 (32.6%)	368 (29.7%)
Proportion <35 ETDRS letters in study eye				
No	564 (83.2%)	164 (79.6%)	293 (82.3%)	1021 (82.3%)
Yes	114 (16.8%)	42 (20.4%)	63 (17.7%)	219 (17.7%)
Proportion <35 ETDRS letters in both eyes				
No	641 (94.5%)	194 (94.2%)	342 (96.1%)	1177 (94.9%)
Yes	37 (5.5%)	12 (5.8%)	14 (3.9%)	63 (5.1%)
Cataract surgery				
No	677 (99.9%)	206 (100%)	356 (100%)	1239 (99.9%)
Yes	1 (0.1%)	0 (0%)	0 (0%)	1 (0.1%)
DME				
No	662 (97.6%)	152 (73.8%)	330 (92.7%)	1144 (92.3%)
Yes	16 (2.4%)	54 (26.2%)	26 (7.3%)	96 (7.7%)
Vitrectomy				
No	678 (100%)	206 (100%)	356 (100%)	1240 (100%)
Yes	0 (0%)	0 (0%)	0 (0%)	0 (0%)
PRP				
No	651 (96.0%)	199 (96.6%)	345 (96.9%)	1195 (96.4%)
Yes	27 (4.0%)	7 (3.4%)	11 (3.1%)	45 (3.6%)

(b)	Baseline VA <70 (n=345)	Baseline VA >70 (n=895)	Overall (n=1240)
Baseline VA			
Mean (SD)	51.3 (12.3)	78.3 (6.24)	70.8 (14.7)
Median (min, max)	55.0 (10.0, 69.0)	76.0 (70.0, 94.0)	76.0 (10.0, 94.0)
Post-event VA (mean; ETDRS letters)			
Mean (SD)	34.8 (18.2)	65.1 (14.3)	56.7 (20.6)
Median (min, max)	35.0 (0, 61.0)	70.0 (0, 89.0)	61.0 (0, 89.0)
Post-event VA ≥70 ETDRS letters			
VA <70	345 (100%)	378 (42.2%)	723 (58.3%)
VA >70	0 (0%)	517 (57.8%)	517 (41.7%)

Visual acuity (VA) in Early Treatment Diabetic Retinopathy Study (ETDRS) letters shown for patients that lost five or more ETDRS letters at the time of visual loss event.

Specific aetiologies of the VA loss event was queried by identifying treatments carried out within 6 months of the event itself: cataract surgery for cataract; anti-VEGF or macular laser for diabetic macular oedema (DME); vitrectomy for vitreous haemorrhage; and panretinal photocoagulation (PRP) for reactivation of proliferative diabetic retinopathy. Cohort was considered overall and stratified by (a) macular status and (b) baseline visual acuity being <70 ETDRS letters or ≥70. Baseline (first recording of stable proliferative diabetes following panretinal photocoagulation) VA in ETDRS letters shown for overall cohort and substratified by macular status. Mean, median, SD, minimum and maximum values shown. Lens status, as well as, history of vitrectomy or DME is also shown.

### Legal limits of driving

When we consider the DMO status in the fellow eyes, 55.3% had no DMO, 14.1% had active DMO (M1A) and 25% stable DMO (M1S) (data not shown). Maculopathy status was not available in 5.6%. In addition, 66% had PRP treated PDR in the fellow eyes. When both eyes are considered together, 15.6% of this study cohort would not be eligible to meet UK driving standards due to the loss of one line of vision in the study eye (table 3a). Additionally, no patients who presented with baseline VA of <70 letters achieved a final VA of ≥70 letters. Vitrectomised eyes that had lost a line of vision had poorer outcomes with a median VA loss of −17.5 letters (three Snellen lines).

### Probability of losing one Snellen line of vision (ETDRS ≥5 letters)

**Figure 1:**
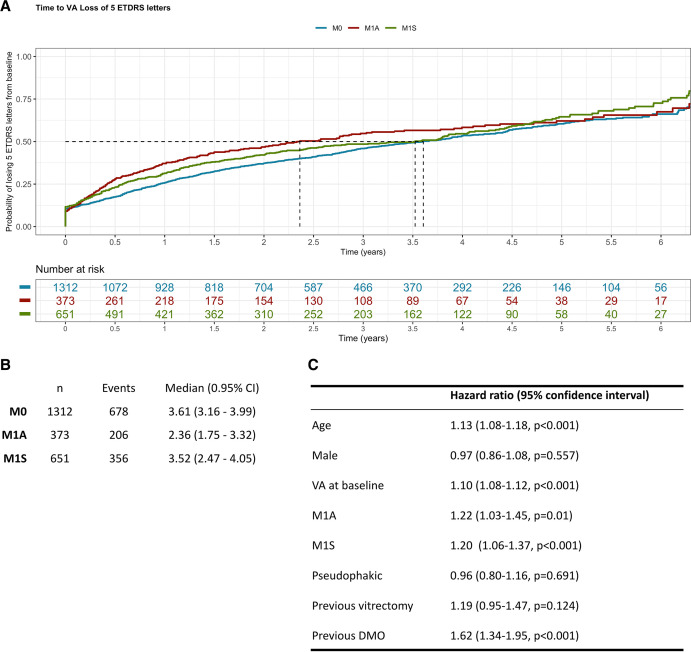
Probability of visual acuity loss of at least five Early Treatment Diabetic Retinopathy Study (ETDRS) letters after achieving stability in proliferative diabetic retinopathy. Kaplan-Meier modelling was carried out to estimate the probability of losing five or more ETDRS letters (Snellen equivalent one line) for stratified by macular status at time of diagnosis. The cohort was substratified by the macular status at baseline (first record of stable proliferative diabetic retinopathy): absence of diabetic macular oedema (DMO; M0), active DMO on treatment (M1A) or stable DMO under observation (M1S). Median time to loss of at least five ETDRS letters was (A) plotted and (B) tabulated comparing each of these subcohorts. (C) Multivariable Cox proportional hazards considering each of the baseline covariables was carried out, including: age, gender, visual acuity (VA), macular status, phakic status, previous vitrectomy, (phakic vs pseudophakic) and previous treatment for DMO.

Kaplan-Meier modelling demonstrates that the median survival time to loss of five ETDRS letters or more was 3.32 (95% CI, 2.94 to 3.78 years, irrespective of the DMO status at baseline (data not shown). Those with active DMO (M1A) at baseline are more likely to experience this event (median event time=2.36 years (95% CI, 1.75 to 3.32)) than those with stable DMO (M1S; 3.52 years (95 CI, 2.47 to 4.05)) and without DMO (M0; 3.61 years (95% CI, 3.16 to 3.99)) (figure 1A, B). A sensitivity analysis removing those who had vitrectomy, repeat PRP or anti-VEGF therapy in the 6 months prior to vision loss did not change these probabilities (data not shown). Multivariable Cox proportional hazards models were used to identify covariates predictive of patients losing five or more ETDRS letters (figure 1C). Increasing age, better baseline VA and coexistent active or stable DMO were predictors of loss of five or more ETDRS letters.

### Evaluating other causes of visual loss

Loss of VA due to cataract, DMO, vitreous haemorrhage, reactivation of proliferative diabetic retinopathy was considered by querying whether cataract surgery, anti-VEGF or macular, vitrectomy and PRP—respectively—had taken place within 6 months of losing five or more ETDRS letters (table 3A). Only 7.7% (96/1240), 3.6% (45/1240) and 0.1% (1/1240) of eyes that reached this event could be explained by DMO, reactivation of proliferative diabetic retinopathy and cataract development, respectively. The data and retinal images of a random sample of 100 patients who lost five or more letters were also evaluated to assess other causes of visual loss such as the presence of clinically significant epiretinal membrane but they were not a major cause of visual loss. There was no reliable records of diabetic macular ischaemia that could be obtained from the electronic medical records as fluorescein angiography, OCT-angiography (OCT-A) or OCT evidence of disorganisation of the inner retina were not systematically recorded.

### Follow-up of stable treated proliferative diabetic retinopathy

**Figure 2:**
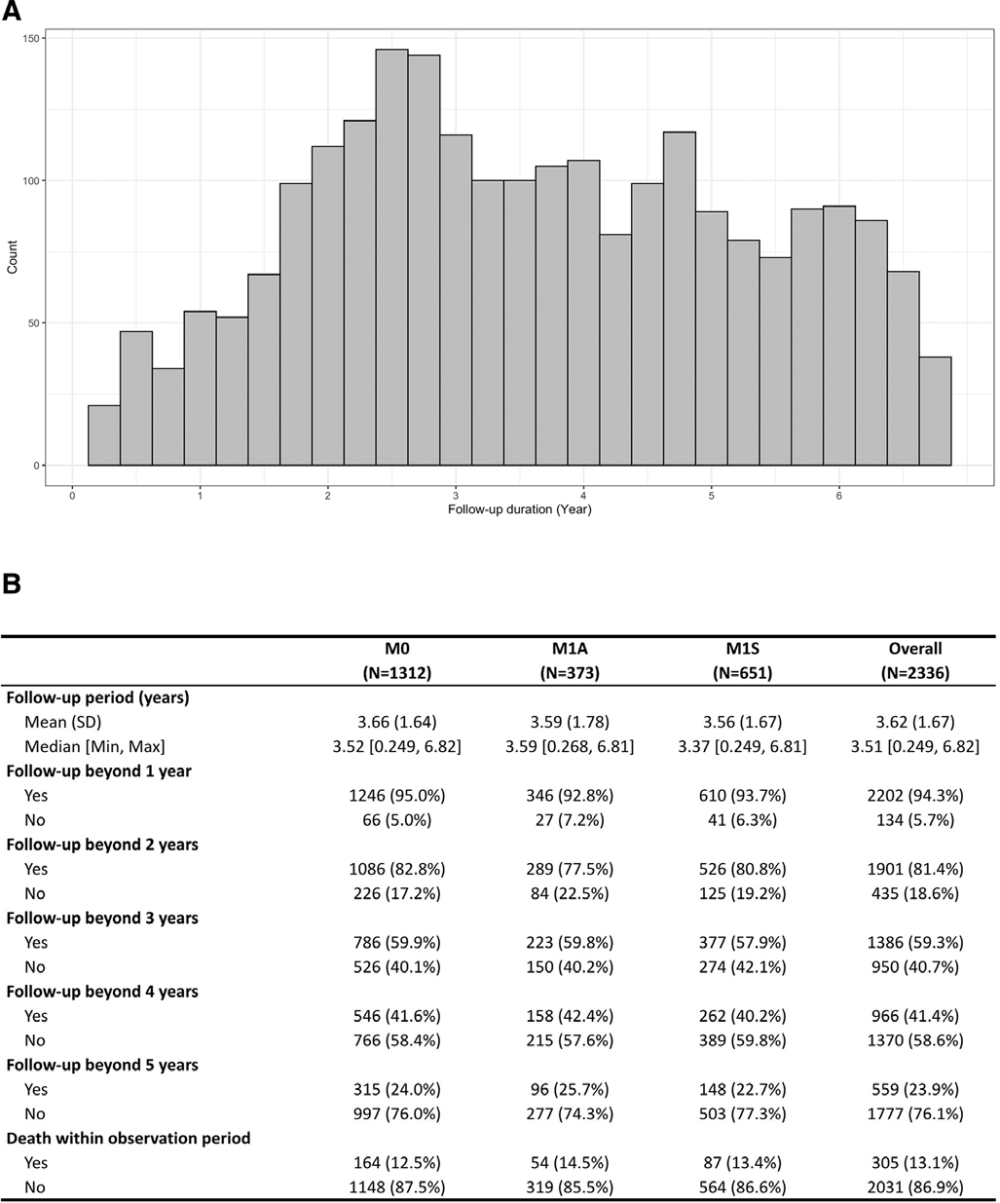
Follow-up and mortality among patients proliferative diabetic retinopathy after being deemed stable (A) Histogram of duration of total follow-up for each patient within the cohort. (B) Mean, median, SD, minimum and maximum follow-up duration in years was queried for the entire cohort (Overall) and the subcohorts stratified by baseline macular status: absence of diabetic macular oedema (DMO; M0), active DMO on treatment (M1A) or stable DMO under observation (M1S). Similarly, the proportion of patients that were followed-up beyond 1 year, 2 years, 3 years, 4 years and 5 years is shown, as well as those with a recorded death within the observation period.

The distribution of last hospital appointments for our patient cohort is shown in figure 2. The mean follow-up was 3.62 (SD 1.67) years and there were no differences between the groups with and without DMO at baseline. Approximately 60% of the cohort had an observation beyond 3 years. However, patients recruited included in the study in 2019 could not have 3 years follow-up and so the follow-up data is better. The mortality rate was about 13% with no differences between groups with and without DMO at baseline.

## Discussion

The results of this retrospective study show that there is a 50% likelihood for a real-world cohort under follow-up after achieving stability post PRP for treatment naïve PDR to maintain their presenting VA for at least 3.5 years. However, in the 50% that lost at least one line of vision, the median loss was nine ETDRS letters, irrespective of the presence of DMO at baseline. A 10-letter loss is a clinically meaningful difference as these changes exceed measurement variability.^23^ In the study, this was corroborated by only taking forward changes present in two consecutive visits. These findings are worse than that reported in the 5-year Protocol S cohort, where 9% lost 10 or more letters from baseline.^14^

A five-letter loss is particularly important in a person whose baseline VA is around 70 letters, when the VA in the other eye is already below 70 letters. Although at baseline, 65% had VA of ≥70 ETDRS letters (Snellen equivalent ≥20/40) in the study eye, only 34% maintained this vision at the point when the study eye lost at least a line of vision, highlighting that stable treated PDR may continue to affect the quality of life of patients. When we consider the VA of both eyes together, this loss of at least one line of vision in the study eye disqualifies approximately 15% of the study cohort from driving based on VA criterion alone.^20^ The baseline predictive factors of 5-letter losers included older age, higher VA and presence of any previous, active or stable DMO. Treating DMO aggressively will likely enable more patients to retain vision that meets the legal limits of driving.

Another important observation noted in the study is the visual outcomes of patients with stable treated PDR with no baseline DMO. Only 70% of these eyes had a VA of 70 letters or better. Other causes of impaired VA in patients with treated PDR include uncorrected refractive errors, visually significant cataract and DMO.^16 24^ Only 11.8% had previous cataract surgery in this study. These figures are similar to the baseline characteristics of the CLARITY trial cohort that was conducted across 22 sites in the UK and the Protocol S study in USA.^12 13^ In addition, 8.5% had previous vitrectomy indicating more advanced PDR and 11.1% had VA of 35 letters or worse (equivalent to Snellen 20/200 and defined as severe visual impairment). Factors that were associated with poor visual outcomes in Protocol S included poor glycaemic control, higher mean arterial pressure, prior DMO and higher severity levels of diabetic retinopathy. Approximately two-thirds of the variations in visual outcome in the trial could not be explained by these associations.^15^ This has been similarly suggested in retrospective analyses (Wykoff *et al*). Although we did not have access to systemic data for our patient cohort, our results also indicate that previous or coexistent DMO is the highest risk for decline in VA in real life. We could not explain why circa 50% of eyes without DMO (676/1312) also experienced a median drop of nine letters. Although 70% of the eyes with no DMO had 70 or more letters at baseline, only 30% of patients in this group had VA of 70 letters or better despite absence of DMO at baseline. New onset DMO that did not meet the central subfield thickness of 400 µm, a requirement to start anti-VEGF in the National Health Service in England and Wales, may be a reason but the database does not capture this data. Other factors such as diabetic macular ischaemia were not quantified by OCT-A in this study. With the advent of OCT-A, it is likely that we will be better informed of its contribution to vision loss in people with PDR. A proportion of patients with PDR also develop disorganisation of the inner retina and this feature is associated with poor visual outcomes.^25^ The inner retinal changes in retinal nerve fibre layer and ganglion cell layer post PRP may also play a role^26 27^ although recent reports suggest that these changes may be age related rather than laser-induced.^28^

Another important finding from this study was the loss to follow-up proportions. Non-English speakers, increasing age, multiple comorbidities and lack of health insurance cover, longer distance travelled for clinical appointment and failure to attend other non-eye-related appointments have all been reported as factors associated with lost to follow-up.^10–12^ Although the proportions of patients with no records increased over time, some patients especially those included from 2019 will have less follow-up and the COVID-19 pandemic could have affected the longer follow-up. However, the mortality rate of 13% is high given that the mean age of this cohort was 59 years. Systematic reviews on this relation have highlighted that increasing severity of diabetic retinopathy is a risk factor for all-cause mortality in both type 1 and 2 diabetes.^29 30^ The findings in this study highlight the need to holistically manage these patients who remain at high risk of visual loss and PDR is a surrogate for generalised vascular dysfunction in diabetes.

This study on a large multiethnic cohort of patients with stable treated PDR in a real-world setting in the National Health Service in the UK is generalisable as the cohort comprises patients with and without concomitant DMO, vitrectomised eyes, both type 1 and 2 diabetes and varying age groups. Due to variability of VA recordings in clinical settings, we mandated 5-letter drop to be confirmed in two consecutive visits. A key assumption implicit to time-event analyses is that censored patients have the same chance of experiencing an event as those still under observation. Survival analyses make use of all available data up until censorship to calculate probabilities of surviving each interval and thereby estimate event probability at any time point. For patients with missing data at a given time point (ie, unknown time to event), the calculated time-event probability can be thought of as a combination of the patient’s own available data and imputation from other patients in their cohort with more data. Another assumption is that time of study enrolment is assumed not to affect event probabilities. It is also important to note that Cox modelling can identify associations between variables.

Limitations of observational studies of real-world clinical data are well recognised.^3^ They inherently feature variability and heterogeneity in patient management, follow-up and outcome measurement. As the Moorfields clinical practice is based on diabetic retinopathy and DMO protocols and treatment is free to all, we believe some of these confounders and bias may be reduced. Nevertheless, other variables such as VA recording in clinical settings may be of concern despite the requirement of two consecutive VA records to confirm five or more letter loss. The proportions of patients lost to follow-up after initial treatment of PDR is also an important discussion point since the evidence of clinical effectiveness of anti-VEGF therapy for PDR were reported.^5–7^ Even in a clinical trial setting, only 60% of the patients were retained by 5 years in Protocol S.^14^ Our study also highlights the increased risk of mortality in patients with PDR. Despite these limitations, the study has enabled hypothesis generation that diabetic macular ischaemia may be a contributory cause for deterioration of VA and that treated PDR may not be a stable disease in some individuals.

10.1136/bmjophth-2022-001068.supp2 Supplementary data

## Data Availability

Data are available upon reasonable request. Data available as per Terms of Data Sharing Agreement within the Data Access Request Service outlined by NHS Digital.
