# Supplementary figures and images for: Visual acuity outcome of stable proliferative diabetic retinopathy following initial complete panretinal photocoagulation
Source: BMJ Open Ophthalmol. 2022 Sep 29;7(1):e001068. doi: 10.1136/bmjophth-2022-001068 (PMC9528610; doi:10.1136/bmjophth-2022-001068)

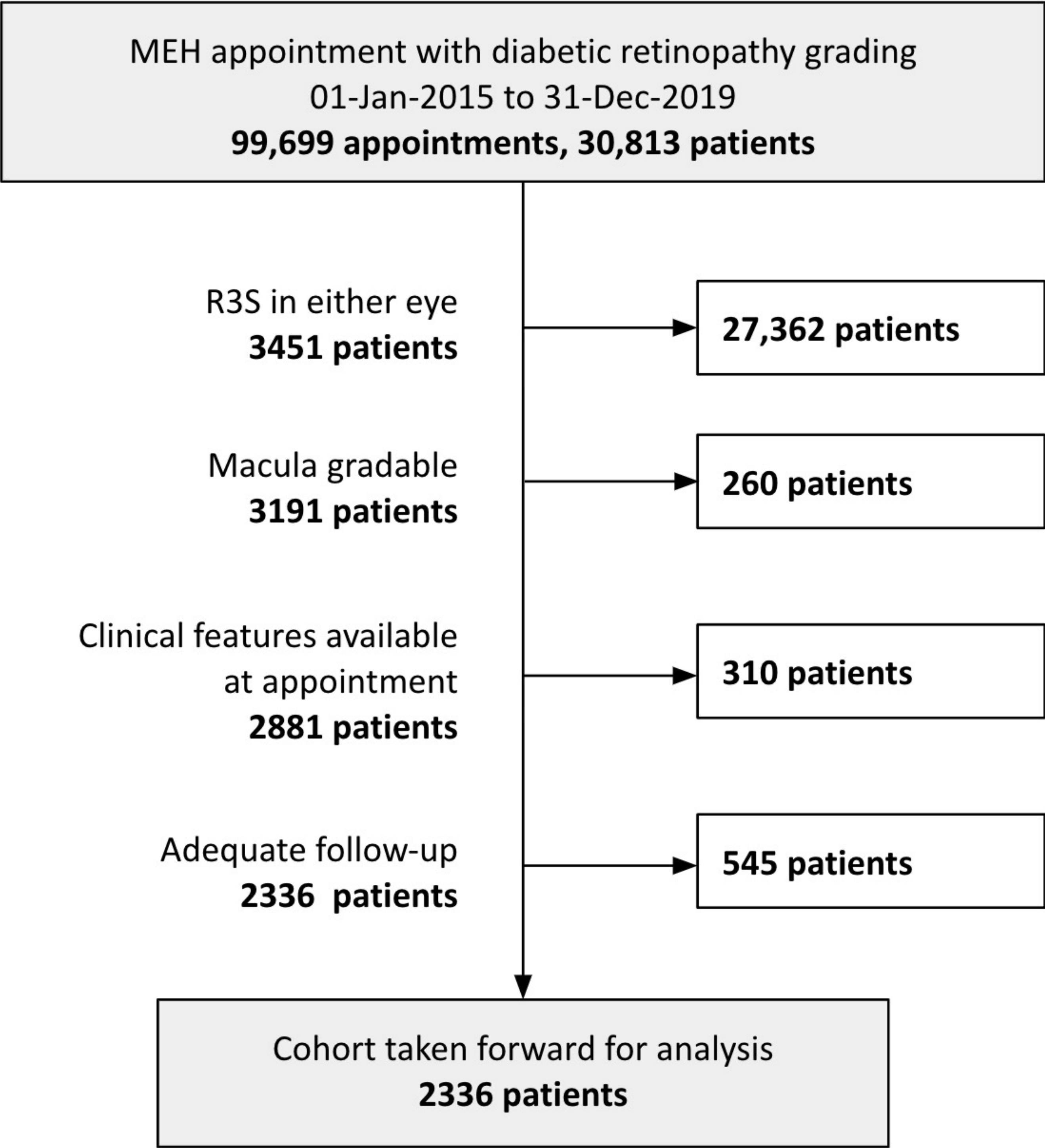

Supplement: Supplementary data [file bmjophth-2022-001068supp001.pdf]
