# Supplementary material for: Visual acuity outcome of stable proliferative diabetic retinopathy following initial complete panretinal photocoagulation
Source: BMJ Open Ophthalmol. 2022 Sep 29;7(1):e001068. doi: 10.1136/bmjophth-2022-001068 (PMC9528610; doi:10.1136/bmjophth-2022-001068)
Supplement: Supplementary data [file bmjophth-2022-001068supp002.pdf]

	M0 (N=1312)	M1A (N=373)	M1S (N=651)	Overall (N=2336)
**Fellow eye Retinopathy**				
R0	39 (3.0%)	9 (2.4%)	7 (1.1%)	55 (2.4%)
R1	91 (6.9%)	27 (7.2%)	44 (6.8%)	162 (6.9%)
R2	205 (15.6%)	94 (25.2%)	122 (18.7%)	421 (18.0%)
R3A	227 (17.3%)	66 (17.7%)	94 (14.4%)	387 (16.6%)
R3S	676 (51.5%)	165 (44.2%)	347 (53.3%)	1188 (50.9%)
U	74 (5.6%)	12 (3.2%)	37 (5.7%)	123 (5.3%)
**Fellow eye Macula**				
M0	1033 (78.7%)	110 (29.5%)	148 (22.7%)	1291 (55.3%)
M1A	74 (5.6%)	182 (48.8%)	74 (11.4%)	330 (14.1%)
M1S	130 (9.9%)	67 (18.0%)	387 (59.4%)	584 (25.0%)
U	75 (5.7%)	14 (3.8%)	42 (6.5%)	131 (5.6%)
**Fellow eye laser**				
No	390 (29.7%)	150 (40.2%)	192 (29.5%)	732 (31.3%)
Yes	893 (68.1%)	219 (58.7%)	442 (67.9%)	1554 (66.5%)
Missing	29 (2.2%)	4 (1.1%)	17 (2.6%)	50 (2.1%)

Supplementary Table 1. Diabetic grading and status of fellow eye at first diagnosis of stable proliferative diabetic retinopathy in study eye
